
JOURNAL INFORMATION
==============================
NLM Title Abbreviation: Wirtschaftsdienst
Journal ID: Wirtschaftsdienst
NLM Journal ID: 101086612

Wirtschaftsdienst (Hamburg, Germany : 1949)

ISSN: 0043-6275

ARTICLE INFORMATION
==============================
PMCID: PMC7964519
PMID: 33746298
DOI: 10.1007/s10273-021-2859-8
Article ID: 2859
Article version: 1
Subjects: Leitartikel

Bildungsverluste durch Corona: Wie lassen sie sich aufholen?

Wößmann Ludger woessmann@ifo.de

grid.469877.3 0000 0004 0397 0846 ifo Zentrum für Bildungsökonomik, ifo Institut, Poschingerstr. 5, 81679 München, Deutschland
Electronic publication date: 2021 Jun 23
Print publication date: 2021
Volume: 101
Issue: 3
First page: 150
Last page: 151
Copyright: © Der/die Autor:in(nen) 2021
License: Open Access: Dieser Artikel wird unter der Creative Commons Namensnennung 4.0 International Lizenz veröffentlicht (https://creativecommons.org/licenses/by/4.0/deed.de).
Open Access wird durch die ZBW — Leibniz-Informationszentrum Wirtschaft gefördert.
License URL: https://creativecommons.org/licenses/by/4.0/

issue-copyright-statement: © The Author(s) 2021

==============================

Seit Mitte Dezember 2020 ist der Großteil der Schüler:innen wieder im Homeschooling. Zwar sind die Grundschüler:innen in den meisten Bundesländern seit wenigen Wochen zumindest abwechselnd im Unterricht, aber ab Klasse fünf dürfen vielerorts nur die Abschlussklassen zur Schule. Zusammen mit dem Lockdown im Frühjahr 2020 fand für viele schon 20 Wochen kein Präsenzunterricht statt – mehr als die Hälfte eines sonst 38 Unterrichtswochen umfassenden Schuljahrs. Ein Ende der Schulschließungen für alle Schüler:innen ist nicht in Sicht. Und da es für sie noch keinen zugelassenen Impfstoff gibt, dürften sich die Probleme auch ins nächste Schuljahr ziehen.

Über die Höhe der entstandenen Bildungsverluste liegen für Deutschland keine konkreten Daten vor. Eine Befragung von über 1.000 Eltern von Schulkindern im ersten Lockdown hat ergeben, dass sich die tägliche Lernzeit der Schüler:innen mehr als halbiert hat, von 7,4 Stunden vor Corona auf 3,6 Stunden während der Schulschließungen (Wößmann et al., 2020). Über ein Drittel hat höchstens zwei Stunden am Tag gelernt. Weit über die Hälfte hatte weniger als einmal pro Woche Online-Unterricht, lediglich 6 % täglich. Noch seltener kam es zu individuellen Kontakten mit den Lehrkräften. Besonders stark war der Rückgang der Lernzeit bei leistungsschwächeren Schüler:innen, denen das selbstständige Lernen ohne die Unterstützung der Lehrkräfte besonders schwerfällt (Grewenig et al., 2020). Sie haben täglich durchschnittlich 6,3 Stunden mit Computerspielen, Handy und Fernsehen verbracht – weit mehr als mit Schule! Wie viel die Kinder im Homeschooling lernen, hängt von der Effektivität ab, die sich je nach Schüler:in stark unterscheiden dürfte. Allerdings wurden die Lernanreize durch die Aussetzung von Prüfungsleistungen deutlich gesenkt. Zudem bedeutet Stillstand Rückschritt: Wenn das Erlernte nicht erprobt und trainiert wird, wird vieles wieder vergessen. Einen Hinweis auf das Ausmaß der Lernverluste gibt eine niederländische Studie. Dort waren die Schulen nur acht Wochen geschlossen, sodass vor den Sommerferien noch die jährlichen nationalen Prüfungen stattfanden. Ihre Auswertung zeigt, dass der Rückgang der Testleistungen im Durchschnitt rund 20 % eines Schuljahrs ausmachte, was eins zu eins der Anzahl der geschlossenen Wochen entspricht (Engzell et al., 2020). Bei Kindern aus bildungsfernen Familien fiel der Lernverlust weit stärker aus. Dass die Schulschließungen bei uns länger andauerten und die Niederlande eine bessere Infrastruktur für digitales Distanzlernen aufweisen, spricht dafür, dass die Lernverluste hierzulande deutlich größer sind. Ökonomisch wird sich das langfristig auswirken: Im Durchschnitt geht das, was man in einem Drittel Schuljahr lernt, im Berufsleben mit rund 3 % höheren Erwerbseinkommen einher (Wößmann, 2020).

Neben den kognitiven Kompetenzverlusten bedeuten die fehlenden Kontakte zu Mitschüler:innen und der dauerhafte Aufenthalt in zum Teil engen Wohnungen für viele auch sozio-emotional eine große Belastung. Fast jedes dritte Kind leidet im zweiten Lockdown unter psychischen Auffälligkeiten (Universitätsklinikum Hamburg-Eppendorf, 2021). Der Anteil mit niedriger gesundheitlicher Lebensqualität ist von 15 % vor Corona auf 40 % während der ersten Welle angestiegen (Ravens-Sieberer et al., 2021). Andererseits wird argumentiert, dass sich das Homeschooling auch positiv auswirken kann, etwa bei den digitalen Kompetenzen, Eigenständigkeit, Selbstorganisation und Resilienz. Angesichts des wenig verbreiteten digitalen Unterrichts und der zunehmenden Belastungen dürften solchen Effekten in der Breite der Schülerschaft aber enge Grenzen gesetzt sein: Bei vielen scheint aus dem anfänglichen Optimismus des ersten Lockdowns mittlerweile Resignation geworden zu sein. Erfahrungen aus früheren Situationen, in denen Schulen länger geschlossen waren, legen nahe, dass die Bildungsverluste nicht gänzlich aufzuholen sind. Beispielsweise wurde für streikbedingte Schulschließungen in Belgien und anderen Ländern gezeigt, dass langfristig niedrigere Bildungsabschlüsse und Einkommen erzielt wurden (Belot und Webbink, 2010; Jaume und Willén, 2019).

Um die Verluste so gering wie möglich zu halten, sollte kurzfristig bei Schulschließungen täglich für alle Schüler:innen verpflichtender Online-Unterricht stattfinden, in dem die Lehrkräfte den Lernstoff per Videokonferenz nach Stundenplan vermitteln. Dass es geht, zeigen derzeit viele Schulen, die sich im Herbst 2020 vorbereitet haben. Die Politik muss klare Vorgaben für flächendeckend verpflichtenden Online-Unterricht machen und die Schulen mit entsprechenden Rahmenregelungen etwa zu funktionsfähiger Videosoftware unterstützen. Dass dies noch nicht geschehen ist, ist schwer verständlich. Denn bloßes Bereitstellen von Arbeitsblättern ist kein Unterricht. Für die Motivation der Schüler:innen ist es wichtig, dass die üblichen Test- und Prüfungsverfahren auch im Distanzunterricht wieder aufgenommen werden. Es hat große Anreizeffekte, ob der Unterrichtsstoff prüfungsrelevant ist oder nicht. Wo immer epidemiologisch möglich, sollten Schulen in den Präsenzunterricht zurückkehren. Dazu sollten wir unsere gesellschaftlichen Prioritäten neu justieren: Andere europäische Länder haben durch weit striktere Einschränkungen für Erwachsene alles darangesetzt, die Schulen offen zu halten. Selbstverständlich sollten in den Schulen alle Schutz- und Hygienemaßnahmen sowie Staffelung von Unterrichtsbeginn und Pausen genutzt werden. Zudem sollten Schüler:innen und Lehrkräfte regelmäßig vor Betreten der Schule Schnelltests machen, um diese zu möglichst sicheren Zonen zu machen. Wo nötig, kann auch Wechselunterricht genutzt werden, wobei entscheidend ist, dass die andere Hälfte der Klasse per Videokonferenz zugeschaltet ist. Schließlich sollte fehlender Stoff durch zusätzliche Fördermaßnahmen nachgeholt werden. Hier sind kreative Lösungen gefragt, vom klassischen Förderunterricht am Nachmittag über Zusatzangebote wie Nachhilfeunterricht, studentische Mentor:innen bis hin zu Ferienprogrammen. Die Unterstützung sollte sich besonders an benachteiligte Schüler:innen richten und auf Basiskompetenzen konzentrieren, die für weiteres Lernen zentral sind. Klassenwiederholungen sollten nur die Ultima Ratio sein. Als besonders effektiv haben sich Mentoringprogramme erwiesen, die Jugendlichen aus stark benachteiligten Verhältnissen jeweils Studierende als Mentor:innen zur Seite stellen (Resnjanskij et al., 2021). So ließe sich Ausgefallenes nicht nur kurzfristig, sondern kontinuierlich nachholen.

Ludger Wößmann leitet das ifo Zentrum für Bildungsökonomik und ist Professor für Volkswirtschaftslehre an der Ludwig-Maximilians-Universität München.


Literatur

Belot M Webbink D Do Teacher Strikes Harm Educational Attainment of Students? LABOUR 2010 24 4 391 406 10.1111/j.1467-9914.2010.00494.x
Engzell, P., A. Frey und M. Verhagen (2020), Learning Inequality during the COVID-19 Pandemic, Mimeo, University of Oxford.
Grewenig, E., P. Lergetporer, K. Werner, L. Wößmann und L. Zierow (2020), COVID-19 and Educational Inequality: How School Closures Affect Low- and High-Achieving Students, CESifo Working Paper, 8648.10.1016/j.euroecorev.2021.103920 PMC8474988 34602646
Jaume D Willén A The Long-Run Effects of Teacher Strikes: Evidence from Argentina Journal of Labor Economics 2019 37 4 1097 1139 10.1086/703134
Ravens-Sieberer, U., A. Kaman, M. Erhart, J. Devine, R. Schlack und C. Otto (2021), Impact of the Covid-19 Pandemic on Quality of Life and Mental Health in Children and Adolescents in Germany, European Child & Adolescent Psychiatry, forthcoming.10.1007/s00787-021-01726-5 PMC7829493 33492480
Resnjanskij, S., J. Ruhose, S. Wiederhold und L. Wößmann (2021), Can Mentoring Alleviate Family Disadvantage in Adolescence? A Field Experiment to Improve Labor-Market Prospects, CESifo Working Paper, 8870.
Universitätsklinikum Hamburg-Eppendorf (2021), COPSY-Studie: Kinder und Jugendliche leiden psychisch weiterhin stark unter Corona-Pandemie, Pressemitteilung, 10. Februar.
Wößmann L Folgekosten ausbleibenden Lernens: Was wir über die Corona-bedingten Schulschließungen aus der Forschung lernen können ifo Schnelldienst 2020 73 6 38 44
Wößmann L Freundl V Grewenig E Lergetporer P Werner K Zierow L Bildung in der Corona-Krise: Wie haben die Schulkinder die Zeit der Schulschließungen verbracht, und welche Bildungsmaßnahmen befürworten die Deutschen? ifo Schnelldienst 2020 73 9 25 39
